# Norovirus GII.3[P25] in Patients and Produce, Chanthaburi Province, Thailand, 2022

**DOI:** 10.3201/eid2905.221291

**Published:** 2023-05

**Authors:** Watchaporn Chuchaona, Sarawut Khongwichit, Woraya Luang-on, Sompong Vongpunsawad, Yong Poovorawan

**Affiliations:** Chulalongkorn University, Bangkok, Thailand (W. Chuchaona, S. Khongwichit, S. Vongpunsawad, Y. Poovorawan);; Ministry of Public Health, Nonthaburi, Thailand (W. Luang-on)

**Keywords:** norovirus, viruses, recombinant, patients, produce, foodborne diseases, food safety, diarrhea, variant, gastroenteritis, Chanthaburi Province, Thailand

## Abstract

An increase in acute gastroenteritis occurred in Chanthaburi Province, Thailand, during December 2021‒January 2022. Of the norovirus genotypes we identified in hospitalized patients and produce from local markets, genotype GII.3[P25] accounted for one third. We found no traceable link between patients and produce but found evidence of potential viral intake.

Noroviruses are the leading cause of sporadic and outbreak-associated, acute, nonbacterial gastroenteritis (*1*). They are genetically diverse and are classified into 10 genogroups (GI‒GX) representing >40 genotypes, although most human noroviruses are GI and GII (*2*). Emergence of recombinant strains that have different combinations of the RNA-dependent RNA polymerase (RdRp) and viral protein 1 (VP1) genes can cause upsurge of new infections (*3*). In 2020, during the early months of the COVID-19 pandemic, public health measures resulted in the drastic reduction of norovirus outbreaks (*4*). We report a resurgence of norovirus in Chanthaburi Province, Thailand

During December 2021‒January 2022, local health authorities in Chanthaburi Province contacted the university for assistance in investigating an increase of vomiting and diarrhea requiring hospitalization among healthy adults. Because preliminary findings by local officials over several weeks had not identified an obvious single-infection source, we suspected norovirus because of rapid widespread community infection. Subsequently, we obtained fecal samples from 34 patients for testing with the approval from the Institutional Review Board of Chulalongkorn University (approval no. 549/62).

Because many patients reported dining out at eateries serving uncooked vegetables, health officials suspected produce as a potential source of infection. Therefore, 24 samples of fresh produce (e.g., salad greens, basil, parsley, napa cabbage, and tomato) from open-air markets near the infection cluster were sent from local health officials for testing to determine a potential norovirus source.

We crushed vegetables in 1 mL nuclease-free water before RNA extraction. We used a bag of ice cubes, which we melted and concentrated from 1 L to 1 mL by using an Amicon Centrifugation Filtration Device (Merck Millipore, https://www.emdmillipore.com) before testing. 

After we performed automated viral RNA extraction by using a magLEAD 12 gC Instrument (Precision System Science, https://www.pss.co.jp), we tested for noroviruses by using a real-time reverse transcription PCR (RT-PCR) (*5*). We dual-typed norovirus-positive samples for the RdRp and VP1 genes by using a conventional RT-PCR (*6*). We genotyped Sanger-sequenced nucleotide sequences by using the Norovirus Genotyping Tool (http://www.rivm.nl/mpf/norovirus/typingtool) and deposited them in GenBank (accession nos. OP210707‒54, OP210788‒834, OP218773‒7, and OP218813‒7). We performed phylogenetic analysis by using the maximum-likelihood method and 1,000 bootstrap replicates implemented in MEGA 11 (http://www.megasoftware.net).

A total of 32/34 patients (age range 1–82 years, mean age ±SD 31.4 ±19.7 years) were positive for norovirus; they had GI only (2/32), GII only (23/32), and GI and GII (7/32) infections. We ascertained nucleotide sequences for all 30 GII-positive samples (Table).

**Table:** Detection of GII noroviruses in patients and produce samples, Chanthaburi Province, Thailand, 2022*

VP1	RdRp gene					
	P7	P12	P17	P21	P25	P31
GII.3	2/4	2/ND	1/1	ND	9/1	1/ND
GII.4 Hong Kong	1/ND	ND	ND/1	ND	ND	1/2
GII.4 Sydney	1/ND	ND	ND	ND	1/ND	ND
GII.6	3/5	ND	1/1	ND	ND/1	ND
GII.7	1/ND	ND	ND	ND	ND	ND
GII.17	ND	ND	2/1	ND	ND	ND
GII.21	ND/1	ND	2/ND	2/ND	ND	ND

*Values indicate patient samples/produce samples positive for norovirus (e.g., the GII.3[P7] combination was detected in 2 patient and 4 produce samples). Several VP1 and RdRp combinations are detected in patient and produce samples. Produce samples were obtained from open-air markets near the infection cluster and sent by local health officials for testing to determine a potential norovirus source. ND, not detected; P, polymerase; RdRp, RNA-dependent RNA polymerase; VP, viral protein.

Analysis of the RdRp gene identified GII.P25 (10/30), GII.P7 (8/30), GII.P17 (6/30), and 2 each of GII.P12, GII.P21, and GII.P31 (Appendix Figure). Analysis of the VP1 gene identified GII.3 (15/30), GII.6 (4/30), GII.21 (4/30), GII.17 (2/30), GII.4 Sydney (2/30), GII.4 Hong Kong (2/30), and GII.7 (1/30). Defined genotypes were GII.6[P7] (3/30); 2 each of GII.3[P7], GII.3[P12], GII.17[P17], GII.21[P17], and GII.21[P21]; and 1 each of GII.3[P17], GII.3[P31], GII.4 Sydney[P7], GII.4 Sydney[P25], GII.4 Hong Kong [P7], GII.4 Hong Kong [P31], GII.6[P17], and GII.7[P7]. We also observed the relatively rare GII.3[P25] genotype (9/30) (*7*).

Testing for a possible source showed that 8/24 produce samples and ice were norovirus-positive; GII.3[P25] was identified in a tomato (Appendix Table). Partial RdRp genes and entire VP1 genes showed closest phylogeny with unpublished GenBank sequences OL451532 and OL451533, which were deposited by health authorities in China during November 2021. GII.3[P25] from Thailand and China clustered away from global strains (Figure).

**Figure:**
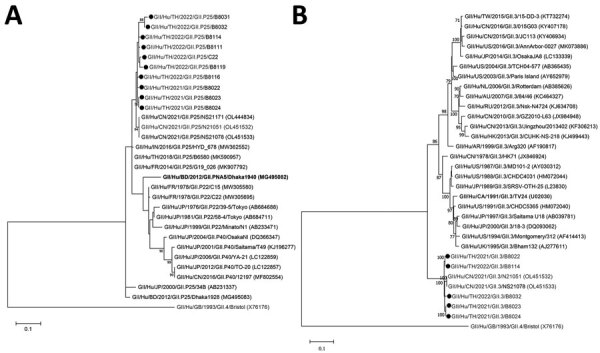
Phylogenetic analysis of norovirus strains, Chanthaburi Province, Thailand, 2022. A) Partial sequence of the RNA-dependent RNA polymerase gene (187 bp). B) Complete sequence of the capsid gene (1,644 bp). Strains identified in this study (black circles) were compared with the reference (bold) and global strains. GenBank accession numbers for strains are indicated in parentheses. Trees were generated by using the maximum-likelihood method with 1,000 bootstrap replicates implemented in MEGA 11 (https://www.megasoftware.net). Bootstrap values >70 are indicated at the nodes. Only strains of sufficient nucleotide sequence length needed for analysis are included. Scale bars indicate nucleotide substitutions per site.

Although only 5 GII.3[P25] strains from Thailand yielded full-length VP1 sequences, deduced amino acid residues in the P2 domain (residues 385–420) were possible for all 10 strains. Alignments showed residue changes D388N, Q391M, N404T, E405D, S412I, N415R, and F420V compared with the prototypic GII.3/TV24 (GenBank accession no. U02030) and more recent GII.3 VP1 strains.

Many different norovirus genotypes found in samples from patients during this investigation did not implicate an overwhelmingly predominant strain responsible for the infection cluster. However, emergence of GII.3[P25] in Thailand identified in patients and produce (sample C22) indicated a potential source of infection. The diversity of norovirus strains in produce sampled warrants increased awareness of food safety in preventing norovirus infection. In addition, we identified GII.4 Hong Kong [P31] and 2 novel variants, GII.4 Hong Kong [P7] (patient B8045) and GII.4 Hong Kong [P17] (sample C30), which were reported recently (*8**,**9*), and GII.21[P17], previously reported in South Korea (*10*).

Combined investigation of illness in patients and of potential sources of infection is often challenging. A limitation of our study was low viral loads (cycle threshold >30) for many of the samples, which hindered confirmation of minor recombinants found. Our study was also limited by the lack of a definitive traceable link between patients and produce but does provide evidence of potential ingestion of the virus. Although contaminated fruits and vegetables can serve as a source of outbreaks in countries in temperate zones, this study paralleled similar transmission, but in a tropical country. Continuous molecular and epidemiologic surveillance of emerging norovirus variants is needed to detect future outbreaks.

Appendix Additional information on norovirus GII.3[P25] in patients and produce, Chanthaburi, Thailand, 2022.
