# Supplementary material for: Norovirus GII.3[P25] in Patients and Produce, Chanthaburi Province, Thailand, 2022
Source: Emerg Infect Dis. 2023 May;29(5):1067–70. doi: 10.3201/eid2905.221291 (PMC10124668; doi:10.3201/eid2905.221291)
Supplement: Appendix — Additional information on norovirus GII.3[P25] in patients and produce, Chanthaburi, Thailand, 2022. [file 22-1291-Techapp-s1.pdf]

Article DOI: https://doi.org/10.3201/eid2905.2211291

EID cannot ensure accessibility for Supplemental Materials supplied by authors. Readers who have difficulty accessing supplementary content should contact the authors for assistance.

Norovirus GII.3[P25] in Patients and Produce, Chanthaburi Province, Thailand, 2022

Appendix.

Appendix Table. Ice and produce tested for norovirus

		Detectable	GII genotyping	
Sample code	Description	Ct value	RdRp	VP1
C01	Ice cubes	33.1	P7	GII.3
C10	Salad greens	32.9	P7	GII.6
C11	Salad greens	33.0	P7	GII.6
C12	Thai basil	–	–	–
C13	Cabbage	30.3	P7	GII.6
C14	Napa cabbage	32.7	P25	GII.6
C15	Salad greens	32.3	P7	GII.21
C16	Salad greens	33.0	P31	GII.4 Hong Kong
C17	Celery	–	–	–
C18	Water spinach	32.5	P7	GII.6
C19	Holy basil	–	–	–
C20	Thai basil	33.0	P17	GII.3
C21	Cucumber	32.5	P7	GII.3
C22	Tomato	30.7	P25	GII.3
C23	Daikon radish	31.2	P31	GII.4 Hong Kong
C24	Water spinach	–	–	–
C25	Salad greens	36.0	P7	GII.3
C26	Culantro	–	–	–
C27	Cilantro	35.4	P7	GII.3
C28	Napa cabbage	33.3	P17	GII.6
C29	Chinese broccoli	34.1	P17	GII.17
C30	Diplazium esculentum fern	34.9	P17	GII.4 Hong Kong
C31	Pak choi	–	–	–
C32	Culantro	36.5	P7	GII.6
C33	Water spinach	–	–	–

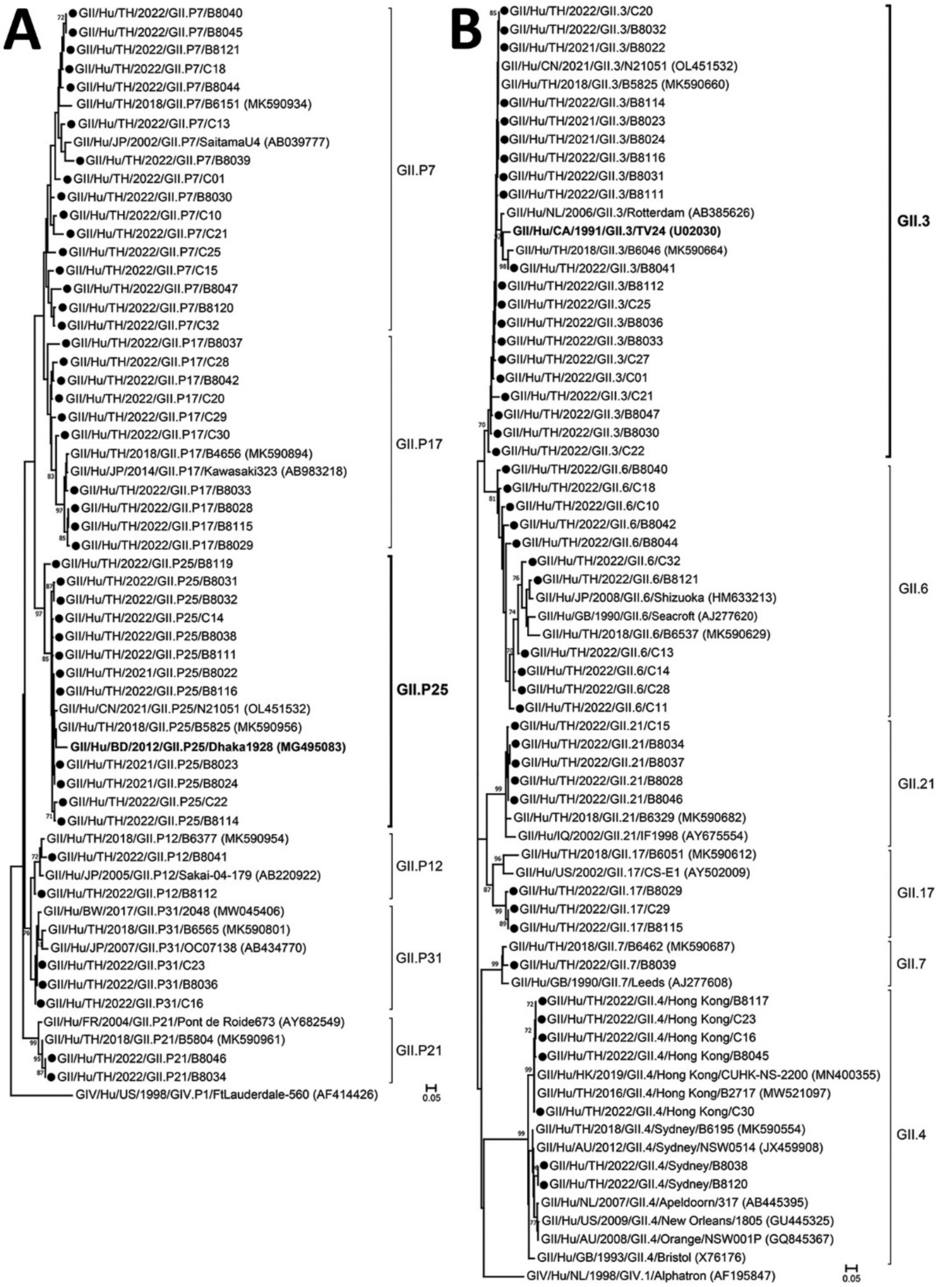

Appendix Figure. Phylogenetic analysis of norovirus strains from Chanthaburi Province, Thailand, 2022. A) Partial sequence of RdRp gene (187 bp). B) Partial sequence of VP1 gene (207 bp). Strains identified in this study (dotted) were compared with the reference (bolded) and global strains. Strain accession numbers are indicated in parentheses. Trees were generated by using the maximum-likelihood method with 1,000 bootstrap replicates implemented in MEGA11 (https://www.megasoftware.net). Bootstrap values $\geq$70 are indicated at the nodes. Scale bars indicate nucleotide substitutions per site.
